# The association between midwifery staffing and reported harmful incidents: a cross-sectional analysis of routinely collected data

**DOI:** 10.1186/s12913-024-10812-8

**Published:** 2024-03-28

**Authors:** Lesley Turner, Jane Ball, Paul Meredith, Ellen Kitson-Reynolds, Peter Griffiths

**Affiliations:** 1 University of Southampton, Southampton, UK; 2 National Institute for Health Research Applied Research Centre (Wessex), Southampton, UK

**Keywords:** Maternity staffing, Skill mix, Workload, Adverse events, Manpower

## Abstract

**Background:**

Independent inquiries have identified that appropriate staffing in maternity units is key to enabling quality care and minimising harm, but optimal staffing levels can be difficult to achieve when there is a shortage of midwives. The services provided and how they are staffed (total staffing, skill-mix and deployment) have been changing, and the effects of workforce changes on care quality and outcomes have not been assessed. This study aims to explore the association between daily midwifery staffing levels and the rate of reported harmful incidents affecting mothers and babies.

**Methods:**

We conducted a cross-sectional analysis of daily reports of clinical incidents in maternity inpatient areas matched with inpatient staffing levels for three maternity services in England, using data from April 2015 to February 2020. Incidents resulting in harm to mothers or babies was the primary outcome measure. Staffing levels were calculated from daily staffing rosters, quantified in Hours Per Patient Day (HPPD) for midwives and maternity assistants. Understaffing was defined as staffing below the mean for the service. A negative binomial hierarchical model was used to assess the relationship between exposure to low staffing and reported incidents involving harm.

**Results:**

The sample covered 106,904 maternal admissions over 46 months. The rate of harmful incidents in each of the three services ranged from 2.1 to 3.0 per 100 admissions across the study period. Understaffing by registered midwives was associated with an 11% increase in harmful incidents (adjusted IRR 1.110, 95% CI 1.002,1.229). Understaffing by maternity assistants was not associated with an increase in harmful incidents (adjusted IRR 0.919, 95% 0.813,1.039). Analysis of specific types of incidents showed no statistically significant associations, but most of the point estimates were in the direction of increased incidents when services were understaffed.

**Conclusion:**

When there is understaffing by registered midwives, more harmful incidents are reported but understaffing by maternity assistants is not associated with higher risk of harms. Adequate registered midwife staffing levels are crucial for maintaining safety. Changes in the profile of maternity service workforces need to be carefully scrutinised to prevent mothers and babies being put at risk of avoidable harm.

**Supplementary Information:**

The online version contains supplementary material available at 10.1186/s12913-024-10812-8.

## Background

Most women in the United Kingdom give birth in hospital, and maternity units also provide antenatal and postnatal inpatient care and specialist services such as day assessment and induction of labour [1]. Predicting the demand for care is challenging, as labour by its nature is unscheduled, and there is great variation in the number of women admitted for labour care on any given day [2]. This poses difficulty in planning the staffing of maternity units, as the goal is to minimise both understaffing and overstaffing of services, as staff resources need to be deployed in a purposeful and efficient way. In some services staffing is planned and reported across a whole maternity unit, and midwives are then deployed on the shift to where they are most needed as part of an escalation process [3]. Across England, staffing levels and composition varies [4], although the National Institute for Health and Care Excellence recommends that an evidence-based tool is used along with other information and professional judgement to help plan staffing levels and skill mix [5]. This must be tailored to the expected demand and acuity of those using the service.

Appropriate staffing of maternity units has been highlighted as a priority, being key to the quality of care provided and affecting the wellbeing of women and babies and their future health [6]. However there is a global shortage of midwives [7] and the education of future midwives takes three years or more in many countries [8]. Additional midwifery workload has become apparent in recent years as midwives now have a wider scope of practice and have taken on some work previously assigned to medical staff [9, 10]. With high vacancy rates, targets have been set to increase the number of midwives in training and reduce the number of staff leaving the profession [11]. Shortages of midwives have led to changes in the services and how they are staffed: reduction of parent education and postnatal care provision from the National Health Service, delegating more activities to maternity care assistants, recruitment of midwives from other countries, closure of birth centres, suspension of continuity of care teams and, in extreme circumstances, the temporary closure of labour wards to new admissions [12]. These changes have been in response to the context rather than being introduced as evidence-based policies and the consequences of service changes are unclear [13].

Birth and the surrounding antenatal and postnatal periods of care are generally safe and most women and babies do not have underlying conditions which put them at risk of complications [14]. However, adverse events do occur and there have been a number of investigations into poor maternity outcomes [15, 16], sparking public concern and media attention. The legal costs and recompense related to maternity cases far exceed those from all other medical specialities [17]. The personal cost to families is immeasurable and distress is increased where events could have been avoided [16]. Women over 40 years, those with medical comorbidity, those from most deprived socio-economic groups, and black and Asian mothers have increased risk of adverse events, including mortality [18].

Staffing is one factor that can be modified, albeit creatively due to the supply issues already described. There is good reason to hypothesise that low staffing levels are linked with maternity adverse events, as several studies in nursing show that low staffing is associated with avoidable harm [19], but far less research relates to maternity care [20]. Health workers have reported that poor staffing levels prevent them from ensuring service user safety [21] and the Francis report into the Mid Staffordshire Trust concluded that many incidents occurred due to short staffing. Psychological research has demonstrated that time pressure is associated with increased mistakes [22], which lends further support to this hypothesis.

Maternity service providers in England are expected to note ‘red flag’ events that can highlight where deficiencies in staffing levels are potentially impacting on the quality of care, allowing shift by shift modifications to be made [5]. Although ‘red flags’ are used in NHS services, we do not have evidence on which indicators are most meaningful or sensitive to staffing input [23]. There are only a small number of research studies in midwifery which examine the association between staffing levels and adverse events [20]. These studies have examined the association between midwifery staffing and perineal trauma, post-partum haemorrhage, birth asphyxia, and admissions to the neonatal unit, with mixed findings [24–29]. The measurement of staffing levels in these studies tended to be aggregated over large populations or time periods. Not all studies had appropriate risk-adjustment, so the impacts of other covariates were not always accounted for when producing an estimate of staffing effects [20]. None of the studies factored in the additional workload which comes from multiple admissions and discharges during a shift, as this activity was not captured alongside the service user census. The problem remains that we are unsure how staffing levels and skill mix influences adverse events for mothers and babies, so optimal and hazardous levels are currently unknown. Staffing interventions should be based on high quality evidence to help predict the effects of changes in staffing and skill mix [13] and avoid adverse events if some of these can be prevented.

The aim of this study is to explore the association between daily midwifery staffing levels and the rate of adverse incidents while controlling for other covariates. This study is urgently needed due to the lack of research evidence in this area and the pace at which policy decisions are being implemented to tackle workforce supply and demand.

## Methods

We conducted a cross-sectional analysis nested within a retrospective longitudinal study [30] in three maternity centres in England. The three centres were selected purposefully to represent different types of services and population demographics to aid the generalisability of findings. Maternity service A is smaller and located within a rural hospital with a local neonatal unit. Maternity services B and C are located within different cities, and both have neonatal intensive care units and fetal medicine facilities. These larger services care for women with more complex pregnancies and serve larger populations.

Ethics permission was gained via the University Ethics Committee ERGO 52,957 and IRAS 128,056. Routine data was extracted for 13th April 2015- 29th February 2020 from incident reporting systems. Incidents were included in the settings of maternity theatres, maternity triage, day assessment units, labour wards, antenatal and postnatal areas. We excluded incidents in neonatal units, community, and freestanding birth centres to retain focus on midwifery staffing and to closely match incidents to rosters for inpatient areas. The incident reports were linked to calendar days as opposed to individual service users. Incidents consist of clinical events along with events involving staff members and equipment. The incident description was recorded by each organisation in two columns and specific incidents were coded if they were recorded in either column, although the data was not supplied in a uniform way across the three organisations. Service user harm classification was extracted from the data. The primary outcome was incidents associated with any harm to mothers or babies. Secondary outcomes were incidents classified as moderate or higher harm, medicines incidents, stillbirth or neonatal death, delay, haemorrhage, third- or fourth-degree tear and incidents relating to discharge.

Staffing data was obtained from electronic rosters for the corresponding time-period. Daily staffing data was obtained from roster systems for 24-hour periods from 07.00 to 07.00. Recorded staffing included permanent, bank and agency staff and all shifts worked, excluding absences. This was matched to the number of incidents occurring on the corresponding calendar day which encompassed most of these working hours. A more precise matching to the exact shift was not possible as the time of the incident was missing in a number of records. Staffing was measured by Hours Per Patient Day (HPPD). The HPPD was calculated for Registered Midwives (RM) and this data included Registered Nurses although there were few (< 0.1%) and they were of a similar grade to midwives. HPPD for Maternity Assistants (MA) were calculated separately. HPPD was calculated by dividing the total worked seconds for the staff group by the ward occupancy, which included both mothers and neonates. Ward occupancy for each 24-hour period was calculated by aggregating the durations of each admitted service user’s stay as recorded to the second in the patient administration systems. Data was cleaned to remove shifts where service user occupancy is zero seconds, and to remove shifts with RM HPPD outliers (defined as RM HPPD < 0.5 or RM HPPD > 48). For staffing variables, we examined staffing levels as low relative to the service mean (“understaffing”) or at/above the mean as a binary variable.

We used a negative binomial model [31] to assess the association between staffing levels and the daily number of incidents, with ward occupancy per day as an offset to account for the varying number of people exposed. The negative binomial model was chosen as it performed better than the Poisson model on the likelihood ratio chi-squared test, and because the incident events were over-dispersed as the variance exceeded the mean. Univariable analyses nested in ward were performed before multivariable analyses.

Model fitting was performed by examining the empty model then adding in staffing data for Registered Midwives (RM) and Maternity Assistants (MA) as variables of interest to the study question. Additional variables were added and removed one at a time and were only retained if they improved model fit by reducing Akaike’s Information Criteria and Bayesian Information Criteria (forward selection). The following variables were tested: weekday or weekend (binary variable), higher than expected admissions plus discharges compared to the mean for each service (binary variable), skill mix as the proportion of registered staff (as a continuous variable), proportion of population over 40 years on each day (continuous variable), and proportion of population with Charlson comorbidity index > 0 (continuous variable). Age over 40 years was chosen as a threshold in the analysis as a higher proportion of women in this age group are at risk of morbidity and mortality, and therefore they represent a vulnerable population [18]. We did not have access to data on population ethnicity to include in the modelling. Checks for collinearity were performed by examining the variance inflation factor. The beta coefficients were exponentiated to report incident rate ratios to aid interpretation of the results.

## Results

During the study period there were a total of 106,904 maternal admissions (see Table 1). Service B accounted for 78,882 (74%) of these admissions. Service A is a smaller service and had 5,754 admissions. Service C had 22,268 admissions and provided a shorter time-span of data, due to data collection system upgrades. Overall, a small number of the population were over 40 years old (3.3%) or had comorbidity (7.0%) on any day, and there was variation between maternity services in this measure. Staffing levels varied between the organisations; mean registered midwife HPPD was 12.7 in service A, 5.0 for service B and 6.9 in service C.

The rate of all reported incidents varied per maternity service, from 10.8 to 24.7 per 100 admissions. Incidents associated with harm varied per service, from 2.1 to 3.0 per 100 admissions. Where harm was rated as at least a moderate classification, the rate ranged from 0.07 to 0.52 incidents per 100 admissions. Data for specific types of incident are shown below in Table 1.

**Table 1:** Descriptive statistics on case mix, staffing and incidents per organisation in the study period

	Maternity A	Maternity B	Maternity C
Dates where data is available	13th April 2015–29th Feb 2020	13th April 2015– 29th Feb 2020	30th March 2016– 29th Feb 2020*
Total number admissions in time period	5,754	78,882	22,268
Proportion of the population > 40 years old averaged across all days	3.8%	3.0%	3.3%
Proportion of the population with any comorbidity averaged across all days	7.2%	8.2%	5.1%
Mean daily staffing levels RM HPPD	12.65	5.02	6.89
Mean daily staffing levels MA HPPD	5.22	2.11	2.58
Number of days out of total where RM staffing was below the mean (expected) level	61%	54%	54%
Average skill mix per day across the service (RM staff hours/RM + MA hours)	0.71	0.69	0.73
**Number of incidents that occurred in each service (per 100 admissions**)			
All incidents in Maternity setting	1423 (24.73)	8524 (10.81)	3108 (13.96)
Incidents associated with any harm	144 (2.50)	1677 (2.13)	667 (3.00)
Incidents rated > = moderate harm	30 (0.52)	52 (0.07)	80 (0.36)
Medicines incident	24 (0.42)	467 (0.59)	351 (1.58)
Stillbirth or neonatal death	30 (0.52)	111 (0.14)	Not available
Any type of delay	129 (2.24)	867 (1.10)	Not available
Haemorrhage	196 (3.41)	856 (1.09)	Not available
Third of fourth degree tear	196 (3.41)	570 (0.72)	Not available
Incident related to discharge	11 (0.19)	234 (0.30)	Not available
Reporting of low staffing or high workload	16 (0.28)	1151 (1.46)	81 (0.36)

*incident data available for shorter time period for maternity service C

## Univariable analyses

Univariable analyses for incidents involving harm are presented in Table 2. Understaffing by midwives (IRR 1.104, 95% CI 1.014, 1.203) and maternity assistants (IRR 1.031, 95% CI 0.947, 1.122) were associated with an increased rate of incidents involving harm, although the finding was only statistically significant for midwives. Higher than expected service user turnover (admissions plus discharges) was associated with a statistically significant increased reporting of harm (IRR 1.203, 95% CI 1.105, 1.309). A higher proportion of the service user population who were over 40 years on a given day, having a more dilute skill mix, weekend versus weekday, and increased proportion of service user comorbidity were not associated with a statistically significant increases in reported harm events (see Table 2).

**Table 2:** Results of univariable analyses for incidents involving harm

	Incident rate ratio for incidents involving harm	95% CI
Understaffing less than the mean for Registered Midwives	1.104	1.014, 1.203
Understaffing less than the mean for Maternity Assistants	1.031	0.947, 1.122
Understaffing less than the mean for Overall staffing (RM + MA)	1.313	0.932, 1.850
Weekend versus weekday	0.943	0.858, 1.036
Higher than expected service user turnover	1.203	1.105, 1.309
Skill mix, proportion of total staff who are Registered	1.661	0.523, 5.273
Proportion of population > 40 years	3.223	0.340, 30.544
Proportion of population with Charlson comorbidity index > 0	0.391	0.077, 1.983

RM Registered Midwife, MA Maternity assistant

## Multivariable analyses

After the model fitting process (see Supplement Table A), the RM understaffing, MA understaffing higher than expected service user turnover and skill mix were retained in the model. Understaffing by registered midwives was associated with an 11% increase in harm incidents and this was statistically significant (adjusted IRR 1.110, 95% CI 1.002, 1.229, Table 3). The effects were in the opposite direction for understaffing by maternity assistants, and not statistically significant (adjusted IRR 0.919, 95% 0.813, 1.039). Having higher than the expected number of admissions and discharges (service user turnover) was associated with an 19% increased risk of harm incidents (adjusted IRR 1.190, 95% CI 1.091, 1.299, statistically significant). Skill mix was associated with an increased risk of harm incidents although the result was not statistically significant and the confidence interval was wide (adjusted IRR 3.101, 95% CI 0.703, 13.677).

**Table 3:** Hierarchical multivariable analysis of staffing below the mean for RM and MA, and the association with harm incidents

Harm Incidents	Incident rate ratio	95% CI
Understaffing RM	1.110	1.002, 1.229
Understaffing MA	0.919	0.813, 1.039
Higher than expected service user turnover	1.190	1.091, 1.299
Skill mix (proportion RM)	3.101	0.703, 13.677

RM Registered Midwife, MA Maternity assistant

## Secondary outcomes

Table 4 reports coefficients for multivariable models using the secondary outcomes of incidents with at least moderate harm, medicines incidents, stillbirth or neonatal death, delay, haemorrhage, and third- or fourth-degree tear. Reports of low staffing or high workload were also studied and compared with the empirical measures of low staffing in HPPD. Full results are reported in supplementary material Tables B–J. Tables K, L of the supplementary material show a breakdown of all incidents by primary descriptor for each of the organisations.

**Table 4:** Results from adjusted models for secondary outcomes

IRR (95% CI)	Registered midwife understaffing	Maternity assistant understaffing	Higher than expected service user turnover
Reports of low staffing / high workload	1.483 (1.273, 1.728)	1.225 (1.015, 1.479)	1.349 (1.183, 1.539)
All incidents	1.007 (0.958, 1.059)	1.058 (0.997, 1.122)	1.176 (1.128, 1.277)
Incidents rated > = moderate harm	1.097 (0.720, 1.670)	1.197 (0.751, 1.909)	1.488 (1.058, 2.091)
Medicines incident	1.018 (0.860, 1.205)	1.032 (0.846, 1.260)	1.042 (0.904, 1.201)
Stillbirth or neonatal death	1.048 (0.688, 1.597)	0.924 (0.559, 1.531)	0.863 (0.601, 1.242)
Any type of delay	1.164 (0.997, 1.360)	0.889 (0.736, 1.075)	1.224 (1.070, 1.401)
Haemorrhage	0.963 (0.822, 1.129)	1.165 (0.961, 1.412)	1.317 (1.148, 1.511)
Third of fourth degree tear	0.846 (0.689, 1.039)	1.247 (0.980, 1.587)	1.459 (1.225, 1.738)
Incident related to discharge	1.113 (0.824, 1.503)	1.053 (0.726, 1.527)	1.512 (1.160, 1.970)

Staffing reported in relation to the mean (under mean vs. at or above mean). RM Registered Midwife, MA Maternity assistant

Incident reports of low staffing or high workload were associated with low staffing (HPPD) for both midwives and maternity assistants, and this finding was statistically significant for both groups. For other secondary outcomes there was an increased risk of most types of incident when registered midwife staffing was below the mean, although none were statistically significant and haemorrhage and perineal tears showed non-significant associations in the opposite direction. Similarly in the majority of analyses of maternity assistant staffing (except stillbirth/neonatal death and delay), the point estimate was in the direction of increased risk of incidents when staffing was below the mean, but not statistically significant.

Higher-than-expected service user turnover (admissions and discharges) had a statistically significant association with secondary outcome incidents in 7 of the 9 analyses in Table 4. The highest point estimate was 1.512 (95% CI 1.160, 1.970) for incidents related to discharge when turnover was higher than the mean.

## Discussion

Almost £2.7 billion pounds was spent by the UK National Health Service in 2022/3 in settling damages and legal costs relating to health care adverse events, highlighting the importance of research and improvement in this area [17]. Our study found evidence of an association between understaffing by registered midwives and an 11% increase in reports of incidents involving harm on days when staffing fell below the mean. In contrast, there was no evidence that low maternity assistant staffing was associated with increased incident rates other than reports of low staffing / high workload. In addition, higher than average service user turnover was associated with increased harm events.

Assuming our estimates represent true associations between understaffing and incidents, it is important to explore the mechanism of this effect. Investigations into maternity underperformance have highlighted poor communication, inadequate working relationships between professionals, lack of risk assessment, failure to escalate and not following guidelines as recurring themes [15, 16]. It is possible that these mechanisms could explain the link between low staffing, poor performance and subsequent adverse events although we did not have the capacity to explore this within our data. Chronic understaffing can also affect attendance at mandatory training which has safety implications.

Our research suggests that it may be harmful to reduce registered midwifery staffing as the estimates point towards an increase in adverse events when understaffing occurs. Solutions need to be found to the ‘midwife exodus’ [32] and vicious circle of low staffing and stretched working conditions which contribute to attrition [33]. Policy makers should exercise caution in substituting unregistered staff to cover midwifery tasks as the effects of this change are not clear and may be detrimental. Our findings contribute to the debate about task shifting in maternity care which has seen the expansion of midwives’ scope of practice [9] and an increased number of tasks undertaken by maternity assistants [34]. The effectiveness and safety of this change has not been confirmed in robust clinical studies and there is no evidence to support this from our study.

One new finding in our research is the consistent and strong association between service user turnover and the increased rates of adverse events in our analyses. This has not been identified within midwifery research before now, although Wilson [3] describes the limitation of using the midnight service user census alone to determine workload, as day attenders are not included. This study did not use a midnight census and yet it still found turnover to have a detrimental effect, which may be explained by the additional tasks and workload involved. High turnover also is likely to affect continuity of care with midwifery staff, which is associated with better outcomes [35]. The shift of staff attention and assimilation of information as women are admitted and discharged creates a greater cognitive burden for staff, and may lead to more errors, omissions, or opportunities for missed communication [36]. As turnover is more variable than the service user census it is harder to plan staffing to accommodate variation, although higher baseline staffing levels are more likely to be able to accommodate periodic increases in demand [37].

The strengths of this study are that we were able to access a large multicentre dataset, daily staffing levels, and a breakdown of incidents by type and severity. This is more granular detail than previous studies and gives a valuable insight into contributing factors to maternity adverse incidents. We were able to adjust for other covariates, so that an assessment of staffing as an independent variable could be obtained. We recognise that the Charlson comorbidity index is not tailored specifically to maternity population and more relevant indices such as the Maternal Comorbidity could have been used, however all indices require further appraisal and this is an evolving area [14]. Our findings are presented for staffing levels relative to the mean, and therefore we are unable to specify the actual staffing levels associated with greater harms. Limitations of this study were the use of observational data and reliance on staff reporting of adverse incidents which is known to be inconsistent among the workforce and likely to represent underreporting [38, 39]. There was a large variation in the total number of incidents that occurred per 100 admissions between each of the maternity services, which could reflect inconsistency in reporting as well as true variation. The cross-sectional nature of this study means that we are unable to imply causality between the exposure to understaffing and subsequent adverse events, although it does provide some suggestive evidence of a link between the two.

Future research using routine data could capture rosters from obstetric and neonatal medical colleagues, as staffing levels in these groups may be associated with adverse events and including them in models may modify the estimates seen for midwives and maternity assistants. Defining understaffing based on planned staffing levels could be considered in addition to mean levels for each service, as this is a more meaningful measure, especially when planned staffing has been calculated to optimise safety and effectiveness in the local population. Where possible, adjustment should be made for advanced maternal age, comorbidity, acuity, ethnic origin as well as population size and turnover within the service. If prospective studies are planned, the recording of adverse events using a predefined guide is advised to improve consistency in reporting this key variable. This would reduce the need for staff judgement on reporting and create a more robust measure. The effects of moving staff from wards to cover shortfalls in other areas should also be explored, both in terms of the safety for the areas they are removed from and the ability of staff to adapt quickly to a new environment partway through their shift. It would also be useful to explore the impact of exposure to understaffing and time-lagged outcomes presenting after discharge.

Further evidence on staffing could also be obtained during maternity investigations and quality monitoring inspections [40]. This contextual information may be important to understand the environment that midwives are working within and additional pressure that they may face during some shifts. Emphasis on routine incident reporting is also important and the reporting of no-harm events has been suggested as a marker of a good safety culture within organisations [41].

In conclusion, we found that midwifery understaffing is associated with an increased rate of harm reporting, and high turnover of service users is an independent risk factor. We cannot use this evidence to imply causality due to the cross-sectional design of this study. Further research is needed to clarify these findings including the contribution of other staff groups such as obstetricians and neonatal staff. Mothers and babies may be at risk of avoidable harm if there are insufficient registered midwives.

### Electronic supplementary material

Below is the link to the electronic supplementary material.

Supplementary Material 1

## Data Availability

Due to the sensitive nature of the data and data sharing agreements with the providers we are unable to freely share the source data, but we guarantee its authenticity and the rigour of methods used in the analysis.

## Abbreviations

HPPD: Hours per Patient Day
IRR: Incident Rate Ratio
CI: Confidence Interval
RM: Registered Midwives
MA: Maternity Assistants
AIC: Akaike’s Information Criteria
BIC: Bayesian Information Criteria
