# Supplementary material for: The association between midwifery staffing and reported harmful incidents: a cross-sectional analysis of routinely collected data
Source: BMC Health Serv Res. 2024 Mar 28;24:391. doi: 10.1186/s12913-024-10812-8 (PMC10976845; doi:10.1186/s12913-024-10812-8)
Supplement: Supplementary file 1 — Supplementary Material 1 [file 12913_2024_10812_MOESM1_ESM.docx]

Supplementary Information

**Table of contents**

[Table A : Model fitting by AIC and BIC values for negative binomial empty model plus the addition of covariates for primary outcome of harmful incidents 2](#_Toc159846350)

[Table B : Secondary analysis with the outcome of any reported incident 3](#_Toc159846351)

[Table C : Secondary analysis with the outcome of incidents rated as moderate harm or above 3](#_Toc159846352)

[Table D : Secondary analysis with the outcome of medicines incidents 3](#_Toc159846353)

[Table E : Secondary analysis with the outcome of stillbirth or neonatal death 4](#_Toc159846354)

[Table F : Secondary analysis with the outcome of delay in care 4](#_Toc159846355)

[Table G : Secondary analysis with the outcome of maternal haemorrhage 4](#_Toc159846356)

[Table H : Secondary analysis with the outcome of third or fourth degree tear 5](#_Toc159846357)

[Table I : Secondary analysis with the outcome of discharge incident 5](#_Toc159846358)

[Table J : Secondary analysis with the outcome of reported low staffing or high workload 5](#_Toc159846359)

[Table K : Breakdown of all incidents Service A 6](#_Toc159846360)

[Table L : Breakdown of all incidents Service B 7](#_Toc159846361)

[Table M : Breakdown of all incidents Service C 8](#_Toc159846362)

##### Table A : Model fitting by AIC and BIC values for negative binomial empty model plus the addition of covariates for primary outcome of harmful incidents

| Analysis number | Model tested | AIC | BIC | Decision |
| --- | --- | --- | --- | --- |
| 1 | Empty model | 8232.344 | 8251.853 |  |
| 2 | 1 adding RM staffing | 8229.155 | 8255.167 |  |
| 3 | 1 adding RM and  MA staffing | **8231.079** | **8263.593** | Keep both RM and MA in full model as variables of interest. Use these AIC/BIC to test adding other variables against one at a time |
| Line 3 was used as a comparison as it included the staffing variables of interest. The aim was to select a model with the lowest AIC and BIC and a difference of 2 units was used as a threshold for improved model fit | | | | |
| 4 | 3 adding weekend/weekday | 8230.641 | 8269.658 | Do not include weekend/weekday in full model as does not improve model fit |
| 5 | 3 adding Turnover | 8216.777 | 8255.794 | Keep turnover in full model as improves model fit |
| 6 | 3 adding Skill mix | 7968.274 | 8006.789 | Keep skill mix in full model as improves model fit |
| 7 | 3 adding proportion age over 40 yrs | 8231.867 | 8270.885 | Do not include age over 40 in full model as does not improve model fit |
| 8 | 3 adding Charlson comorbidity | 8231.831 | 8270.849 | Do not include comorbidity in full model as does not improve model fit |
| 9 | Full model including RM staffing, MA staffing, Turnover and Skill mix | 7954.899 | 7999.833 | Coding for full model* |

*_menbreg Any_harm_incident undermeanstaffRM undermeanstaffMA Higher_expectedturnover proportion_RM, exposure(mean_pat_days_org) || organisation:, allbaselevels irr

##### Table B : Secondary analysis with the outcome of any reported incident

| Any_incidents | IRR Std. Err. z P>\|z\| [95% Conf. Interval] |
| --- | --- |
| undermeanstaffRM | 1.007 0.026 0.27 0.783 0.958 1.059 |
| undermeanstaffMA | 1.058 0.032 1.86 0.063 0.997 1.122 |
| Higher_expectedturnover | 1.176 0.025 7.51 0.000 1.128 1.227 |
| proportion_RM | 0.828 0.298 -0.52 0.601 0.409 1.677 |
| _cons | 0.040 0.012 -11.04 0.000 0.023 0.071 |
| ln(mean_pat_days_org) | 1.000 (exposure) |
| /lnalpha | -2.182 0.091 -2.360 -2.004 |
| var(_cons) | 0.063 0.052 0.013 0.320 |

##### Table C : Secondary analysis with the outcome of incidents rated as moderate harm or above

| harm_moderateplus | IRR Std. Err. z P>\|z\| [95% Conf. Interval] |
| --- | --- |
| undermeanstaffRM | 1.097 0.235 0.43 0.667 0.720 1.670 |
| undermeanstaffMA | 1.197 0.285 0.76 0.450 0.751 1.909 |
| Higher_expectedturnover | 1.488 0.258 2.29 0.022 1.058 2.091 |
| proportion_RM | 0.146 0.437 -0.64 0.520 0.000 50.900 |
| _cons | 0.002 0.003 -3.01 0.003 0.000 0.103 |
| ln(mean_pat_days_org) | 1.000 (exposure) |
| /lnalpha | 0.415 0.613 -0.786 1.615 |
| var(_cons) | 0.680 0.577 0.129 3.588 |

##### Table D : Secondary analysis with the outcome of medicines incidents

| medicines_incident | IRR Std. Err. z P>\|z\| [95% Conf. Interval] |
| --- | --- |
| undermeanstaffRM | 1.018 0.088 0.21 0.837 0.860 1.205 |
| undermeanstaffMA | 1.032 0.105 0.31 0.755 0.846 1.260 |
| Higher_expectedturnover | 1.042 0.076 0.56 0.572 0.904 1.201 |
| proportion_RM | 1.082 1.373 0.06 0.951 0.090 13.013 |
| _cons | 0.002 0.002 -6.64 0.000 0.000 0.011 |
| ln(mean_pat_days_org) | 1.000 (exposure) |
| /lnalpha | -1.649 0.582 -2.790 -0.508 |
| var(_cons) | 0.358 0.309 0.066 1.944 |

##### Table E : Secondary analysis with the outcome of stillbirth or neonatal death

| stillbirthornnd | IRR Std. Err. z P>\|z\| [95% Conf. Interval] |
| --- | --- |
| undermeanstaffRM | 1.047912 .2251551 0.22 0.828 .6877575 1.596668 |
| undermeanstaffMA | .9244814 .238015 -0.30 0.760 .5581483 1.531252 |
| Higher_expectedturnover | .8638948 .1600263 -0.79 0.430 .6008774 1.242041 |
| proportion_RM | .0552131 .1597032 -1.00 0.317 .0001905 16.00052 |
| _cons | .0056749 .0115587 -2.54 0.011 .0001048 .3073845 |
| ln(mean_pat_days_org) | 1 (exposure) |
| /lnalpha | .7855324 .4598692 -.1157946 1.686859 |
| var(_cons) | .3853879 .4114015 .0475599 3.122882 |

##### Table F : Secondary analysis with the outcome of delay in care

| delay | IRR Std. Err. z P>\|z\| [95% Conf. Interval] |
| --- | --- |
| undermeanstaffRM | 1.164 0.092 1.92 0.055 0.997 1.360 |
| undermeanstaffMA | 0.889 0.086 -1.22 0.224 0.736 1.075 |
| Higher_expectedturnover | 1.224 0.084 2.95 0.003 1.070 1.401 |
| proportion_RM | 3.017 3.330 1.00 0.317 0.347 26.247 |
| _cons | 0.002 0.001 -8.31 0.000 0.000 0.007 |
| ln(mean_pat_days_org) | 1.000 (exposure) |
| /lnalpha | -1.548 0.351 -2.237 -0.859 |
| var(_cons) | 0.053 0.059 0.006 0.470 |

##### Table G : Secondary analysis with the outcome of maternal haemorrhage

| haemorrhage | IRR Std. Err. z P>\|z\| [95% Conf. Interval] |
| --- | --- |
| undermeanstaffRM | 0.963 0.078 -0.46 0.644 0.822 1.129 |
| undermeanstaffMA | 1.165 0.114 1.55 0.121 0.961 1.412 |
| Higher_expectedturnover | 1.317 0.092 3.93 0.000 1.148 1.511 |
| proportion_RM | 1.567 1.742 0.40 0.686 0.177 13.840 |
| _cons | 0.003 0.002 -7.31 0.000 0.001 0.013 |
| ln(mean_pat_days_org) | 1.000 (exposure) |
| /lnalpha | -1.045 0.260 -1.555 -0.536 |
| var(_cons) | 0.152 0.157 0.020 1.147 |

##### Table H : Secondary analysis with the outcome of third or fourth degree tear

| third_fourthtear | IRR Std. Err. z P>\|z\| [95% Conf. Interval] |
| --- | --- |
| undermeanstaffRM | 0.846 0.089 -1.60 0.111 0.689 1.039 |
| undermeanstaffMA | 1.247 0.153 1.80 0.072 0.980 1.587 |
| Higher_expectedturnover | 1.459 0.130 4.23 0.000 1.225 1.738 |
| proportion_RM | 0.277 0.377 -0.94 0.346 0.019 4.003 |
| _cons | 0.008 0.009 -4.48 0.000 0.001 0.066 |
| ln(mean_pat_days_org) | 1.000 (exposure) |
| /lnalpha | 0.345 0.140 0.070 0.620 |
| var(_cons) | 0.555 0.560 0.077 4.010 |

##### Table I : Secondary analysis with the outcome of discharge incident

| discharge | IRR Std. Err. z P>\|z\| [95% Conf. Interval] |
| --- | --- |
| undermeanstaffRM | 1.113 0.171 0.70 0.484 0.824 1.503 |
| undermeanstaffMA | 1.053 0.200 0.27 0.785 0.726 1.527 |
| Higher_expectedturnover | 1.512 0.204 3.06 0.002 1.160 1.970 |
| proportion_RM | 0.058 0.127 -1.30 0.193 0.001 4.201 |
| _cons | 0.004 0.006 -3.71 0.000 0.000 0.075 |
| ln(mean_pat_days_org) | 1.000 (exposure) |
| /lnalpha | -1.066 0.910 -2.848 0.717 |
| var(_cons) | 0.000 0.000 . . |

##### Table J : Secondary analysis with the outcome of reported low staffing or high workload

| staffing_incident | IRR Std. Err. z P>\|z\| [95% Conf. Interval] |
| --- | --- |
| undermeanstaffRM | 1.483 0.116 5.05 0.000 1.273 1.728 |
| undermeanstaffMA | 1.225 0.118 2.11 0.035 1.015 1.479 |
| Higher_expectedturnover | 1.349 0.091 4.46 0.000 1.183 1.539 |
| proportion_RM | 2.286 2.540 0.74 0.457 0.259 20.182 |
| _cons | 0.000 0.000 -8.27 0.000 0.000 0.003 |
| ln(mean_pat_days_org) | 1.000 (exposure) |
| /lnalpha | -0.626 0.155 -0.930 -0.322 |
| var(_cons) | 0.833 0.717 0.154 4.503 |

##### Table K : Breakdown of all incidents Service A

(first descriptive column only, incidents >1% of total incidents listed in table )

| Percentage of all reported incidents | |
| --- | --- |
| Simple complication of treatment* | 21.50% |
| Third or fourth degree tears | 13.77% |
| Unexpected admission to Neo-Natal Unit | 4.43% |
| Failure in referral process | 4.08% |
| Labour or delivery – other | 3.37% |
| Post-partum haemorrhage > 1,000ml | 3.16% |
| Delay or failure to monitor | 1.90% |
| Failure to follow up | 1.90% |
| Failure/delay to order correct tests, image etc | 1.90% |
| Diagnostic Images / specimens - mislabelled / unlabelled | 1.83% |
| Patient incorrectly identified | 1.76% |
| Diagnostic images / specimens - inadequate / incomplete | 1.62% |
| Stillbirth | 1.62% |
| Unexpected re-admission or re-attendance | 1.62% |
| Documentation (including records, identification) other | 1.55% |
| Failure to note relevant information in patient's record | 1.55% |
| Delay / difficulty in obtaining clinical assistance | 1.48% |
| Communication failure - outside of immediate team | 1.26% |
| Delay in diagnosis for no specified reason | 1.19% |
| Cord PH < 7.15 | 1.12% |
| Unplanned admission / transfer to specialist care unit | 1.12% |
| Breach of patient confidentiality | 1.05% |
| Failure in booking process | 1.05% |

*when examining second column this was mainly post partum haemorrhage and shoulder dystocia

##### Table L : Breakdown of all incidents Service B

(first descriptive column only, incidents >1% of total listed in table)

| Maternity triggers* | 39.98% |
| --- | --- |
| Communication failure | 7.09% |
| Delay / failure to treatment or procedure | 6.43% |
| Documentation and Health Records | 6.41% |
| Staffing | 6.21% |
| Medication | 5.40% |
| Medical Devices | 3.67% |
| Blood Transfusion | 3.65% |
| Nursing/Midwifery Staffing | 2.87% |
| Access, Admission, Discharge and Transfer issue | 2.37% |
| Sharps incident | 1.82% |
| Diagnosis, Scans and Tests | 1.70% |
| Tissue Viability | 1.24% |
| Aggression, Violence and Harassment | 1.23% |
| Other (Patient-Safety) | 1.22% |

*includes post partum haemorrhage, unplanned neonatal unit admission, shoulder dystocia, third degree tear, low cord pH, low apgar score

##### Table M : Breakdown of all incidents Service C

(first descriptive column only, incidents >1% of total listed in table)

| Clinical Event | 48.22% |
| --- | --- |
| Non Clinical Event | 18.74% |
| Medication | 11.53% |
| Pathology/Blood/Screening | 8.49% |
| Accident including slips, trips and falls | 4.26% |
| Staff Shortage | 2.80% |
| Equipment | 1.31% |
| Tissue Damage | 1.25% |
